# Accuracy and complications of CT-guided pulmonary core biopsy in small nodules: a single-center experience

**DOI:** 10.1186/s40644-019-0240-6

**Published:** 2019-07-23

**Authors:** Ming-De Huang, Hsu-Huei Weng, Sheng-Lung Hsu, Li-Sheng Hsu, Wei-Ming Lin, Chien-Wei Chen, Yuan-Hsiung Tsai

**Affiliations:** 1 Department of Diagnostic Radiology, Chang-Gung Memorial Hospital, Chiayi branch, Chiayi, Taiwan; 2 College of Medicine, Chang Gung University, Taoyuan, Taiwan; 3 Institute of Medicine, Chung Shang Medical University, Taichung, Taiwan

**Keywords:** Lung nodules, CT-guided biopsy, Pneumothorax, Pulmonary hemorrhage

## Abstract

**Background:**

Computed tomography (CT)-guided pulmonary core biopsies of small pulmonary nodules less than 15 millimeters (mm) are challenging for radiologists, and their diagnostic accuracy has been shown to be variable in previous studies. Common complications after the procedure include pneumothorax and pulmonary hemorrhage. The present study compared the diagnostic accuracy of small and large lesions using CT-guided core biopsies and identified the risk factors associated with post-procedure complications.

**Methods:**

Between January 1, 2016, and December 31, 2017, 198 CT-guided core biopsies performed on 195 patients at our institution were retrospectively enrolled. The lesions were separated into group A (< or = 15 mm) and group B (> 15 mm) according to the longest diameter of the target lesions on CT. Seventeen-gauge introducer needles and 18-gauge automated biopsy instruments were coaxially used for the biopsy procedures. The accuracy and complications, including pneumothorax and pulmonary hemorrhage, of the procedures of each group were recorded. The risk factors for pneumothorax and pulmonary hemorrhage were determined using univariate analysis of variables.

**Results:**

The diagnostic accuracies of group A (n = 43) and group B (n = 155) were 83.7 % and 96.8 %, respectively (*p* = 0.005). The risk factors associated with post-biopsy pneumothorax were longer needle path length from the pleura to the lesion (*p* = 0.020), lesion location in lower lobes (*p* = 0.002), and patients with obstructive lung function tests (*p* = 0.034). The risk factors associated with post-biopsy pulmonary hemorrhage were longer needle path length from the pleura to the lesion (*p* < 0.001), smaller lesions (*p* < 0.001), non-pleural contact lesions (*p* < 0.001), patients without restrictive lung function tests (*p* = 0.034), and patients in supine positions (*p* < 0.003).

**Conclusion:**

CT-guided biopsies of small nodules equal to or less than 15 mm using 17-gauge guiding needles and 18-gauge biopsy guns were accurate and safe. The biopsy results of small lesions were less accurate than those of large lesions, but the results were a reliable reference for clinical decision-making. Understanding the risk factors associated with the complications of CT-guided biopsies is necessary for pre-procedural planning and communication.

## Background

Lung cancer is the leading cause of death in all malignancies worldwide [1]. The early detection of lung cancer is important and achievable due to the high accessibility of computed tomography (CT). When lung nodules or masses are suspected to be malignant, the next step is always to obtain the tissue proof. Three tools are feasible to obtain the samples, including transthoracic CT-guided biopsies, endobronchial biopsies (EBB), and video-assisted thoracoscopic (VATS) biopsies. Transthoracic CT-guided biopsies are advantageous for peripheral nodules and prevent the sacrifice of normal lung tissue from lobectomy. Two biopsy methods are used prevalently with CT guidance, fine needle aspiration biopsy (FNAB) and core biopsy. Previous articles [2, 3] noted some advantages of core biopsies over FNAB, including higher diagnostic accuracy of non-malignant samples, better tissue characterization of carcinoma lesions, and better ability to diagnose carcinoma in the absence of pathologists. The diagnostic accuracy of CT-guided core biopsy was 51.4 % to 95.8 % in previous studies [2–16]. However, previous studies primarily focused on larger lesion sizes. Small lesions less than 15 mm remain very challenging for radiologists.

Another concern is the complications of CT-guided biopsy. Pneumothorax and pulmonary hemorrhage are common complications after the procedure. Numerous studies [17–22] investigated factors associated with pneumothorax and pulmonary hemorrhage. Nour-Eldin et al. [17, 18] surveyed the risk factors associated with post-biopsy pneumothorax and pulmonary hemorrhage and found some factors related to pneumothorax and pulmonary hemorrhage, including smaller target nodules, longer needle path length in aerated lung, and middle or lower zone-located nodules. The present study compared the diagnostic accuracy and complication of small and large lesions when performing CT-guided core biopsies and identified the risk factors associated with post-procedure complications, including pneumothorax and pulmonary hemorrhage.

## Methods

### Study population and design

This study was retrospective, and the Institutional Review Board (IRB) approved the study. Between January 1, 2016, and December 31, 2017, 198 CT-guided core biopsies of lung were performed on 195 patients by radiologists whose practice licenses were registered at Chang Gung Memorial Hospital, Chiayi branch, Chiayi, Taiwan. The lesions were further separated into group A and group B according to the longest diameter of target lesions in an axial view on CT with a standard lung window (window level = -600 Hounsfield Units, window width = 1500). If the lesion was equal to or less than 15 mm, it was sorted into group A. If the lesion was larger than 15 mm, it was sorted into group B. One radiologist (Ming-De Huang) retrospectively reviewed the CT images obtained during the procedure. The clinical decision for CT-guided pulmonary biopsy was made via consensus between pulmonologists and radiologists. Patients who were not able to follow the instructions required during the biopsy procedure, including maintaining the same position and breath-holding, were rejected. Informed consent was obtained from the patient or their family, and a CT-guided core biopsy was performed on a scheduled day. Laboratory data, including prothrombin time-to-international normalized ratio (PT-INR) and platelet count, were checked within 3 days before the biopsy procedure. The biopsies were only performed in patients with a PT-INR less than 1.5 and platelet count of at least 5000/mm^3^. Lung function test results were also collected if the patient had performed a lung function test within the 3 months before the biopsy.

### Biopsy procedure

**Fig. 1:**
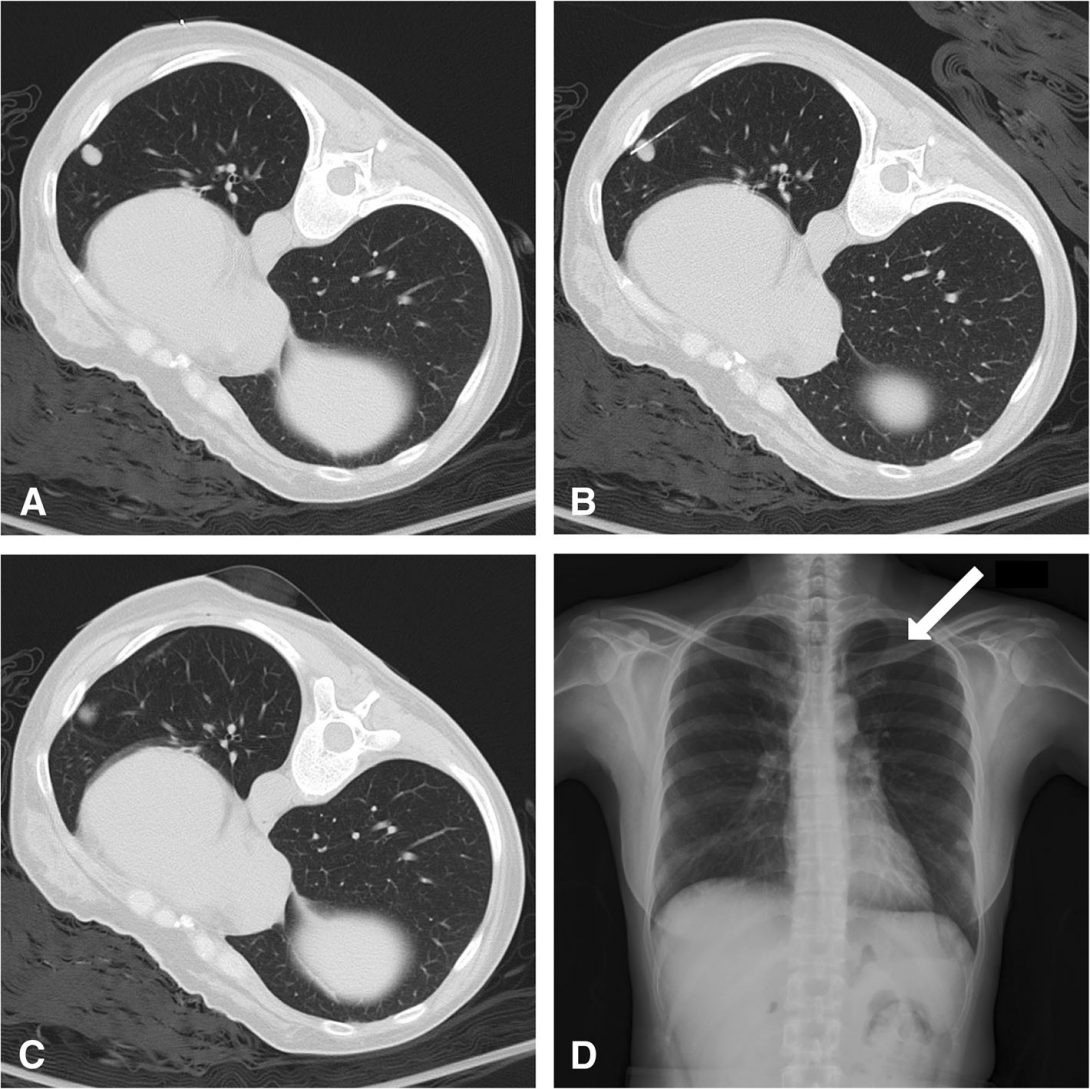
CT-guided pulmonary biopsy in a 45-year-old female. **a** Pre-biopsy CT with standard lung window revealed a 10-mm nodule over the left lower lobe. A metallic marker was placed over the skin for location. **b** The biopsy was performed using a 17-gauge introducing needle and 18-gauge cutting needle. To prevent the needle from crossing the fissure and being blocked by the rib, a proper entry site was chosen. The final position of the cutting notch of the needle was just inside the nodule. **c** Post-biopsy CT revealed minimal pulmonary hemorrhage around the needle path, and no pneumothorax was found. **d** A standing chest X-ray was performed 24 hours after the procedure. The pleural line (arrow) over the left upper chest was visible, and pneumothorax over the left side was confirmed

The biopsy procedures were performed by six radiologists (Bo-Yau Yang with 26 years of experience, Li-Wen Li with 17 years of experience, Sheng-Lung Hsu with 12 years of experience, Li-Sheng Hsu with 10 years of experience, Wei-Ming Lin with 6 years of experience, and Chien-Wei Chen with 3 years of experience). The operator reviewed the pre-biopsy CT images of the patients and decided the patient position. The patient was transferred to the CT table and placed in a supine, prone, or lateral decubitus position. The principles used to decide the puncture route included avoiding obstacles such as the ribs or scapulas, avoiding passing fissures, avoiding pre-existing blebs and bullae, and avoiding vital vasculature. The needle path length from the pleura to the target lesion was also chosen to be as short as possible. After sterile preparation, approximately 10 mL of 2 % lidocaine was injected into the skin for local anesthesia. CT-guided core biopsies were performed using a 64-slice CT (Somatom Sensation 64 CT scanner, Siemens Medical Systems, Erlangen, Germany). Images of 3-mm slice thickness with a standard lung window were acquired during the entire procedure. The biopsies were performed using the coaxial method. A 17-gauge introducer needle (Co-Axial Introducer Needle, Argon Medical Devices, Athens, USA) was introduced first to the edge of the lesion. The inner stylet was removed, and an 18-gauge automated biopsy instrument (SuperCore Biopsy Instrument, Argon Medical Devices, Athens, USA) was introduced through the central canal of the introducer needle coaxially. The specimen notch of biopsy instrument was 19 mm or 9.5 mm, according to the lesion size. Multiple tissue sampling may be achieved using the coaxial method. The number of samples varied and depended on the appearance of the sample and the patient’s condition. All tissue samples were preserved in a 10 % formaldehyde solution and sent to pathological department for further analyses. A last CT scan was performed after the entire procedure to check for the occurrence of pneumothorax or pulmonary hemorrhage. If the patient felt chest discomfort or blood oxygen dropped, which occur during a major pneumothorax, a chest tube was inserted into the pleural cavity. All patients required bed rest for at least 4 hours in the ward after the biopsy. Another chest X-ray was obtained from all patients after 24 hours to follow up on the pulmonary situation (Fig. 1). Pneumothorax was reported based on the evidence either in post-biopsy CT images or in the chest radiographs on the following day. The evidence of pneumothorax included any air densities in the pleural cavity in post-biopsy CT images, or any detectable pleural lines in the chest X-ray on the following day. Pulmonary hemorrhage was reported based on the evidence in post-biopsy CT images. The evidence of pulmonary hemorrhage included peri-lesional, or peri-needle-path ground glass opacity (GGO), or patchy opacity that was not seen in pre-procedure CT images.

### Statistical analyses

The patients’ characteristics, including age, sex, and lung function test results of group A and group B, were recorded. The mean lesion size, the location of the target lesion, and the needle path length between the target lesion and pleura of each group were recorded as lesion variables. The patient position during the procedure, the number of tissues sampled, complications after the procedure (including pneumothorax, chest tube insertion and pulmonary hemorrhage), the radiation dose, and the diagnostic accuracy of each group were recorded as procedure variables. The sensitivity, specificity and diagnostic accuracy of these two groups of lesions were compared. Positive biopsy results were considered a true positive if the pathology results were malignant and in concordance with the surgical results, if the pathology findings were compatible with the patient’s primary malignancy, or if the patient had a clinical course consistent with malignancy. Otherwise, the positive biopsy results were considered a false positive. Negative biopsy results were considered a true negative if the pathology results were benign and further surgical procedures confirmed the benign entities, or if the lesions remained stable or regressed during the 1 years of follow-up. False negative results were considered with further tissue proof of malignancy or if an obviously malignant clinical course was confirmed. Factors hypothesized as associated with pneumothorax and pulmonary hemorrhage were also analyzed, including the needle path length from the pleura to lesion, nodule size, lobe of the lesion, number of samples, presence or absence of pleural contact, lung function test, and patient position during the procedure. All statistical tests were performed using SPSS software (IBM Corp. Released 2011. IBM SPSS Statistics for Windows, Version 20.0. Armonk, NY: IBM Corp. ). Student’s t-test or the Mann-Whitney U test was used for continuous data, depending on the distribution of the data. Fisher’s exact test or the chi-square test was used for binary data depending on the size of the sample. The significance threshold of the *p*-value was set at < 0.05.

## Results

he patient characteristics are listed in Table 1. The two groups of people share the same baseline characteristics, except that lesions in group B are more close to the pleura (distance from the lesion to the pleura, mean ± standard deviation, 16.4 ± 16.3 mm in group B versus 23.2 ± 19.9 mm in groupA, *p*-value = 0.023). The final biopsy results are listed in Table 2. Twelve of the 198 lesions were false negatives, the other 186 lesions were true positive or true negative lesions. The overall accuracy was 93.9 %. The accuracy for each group was 83.7 % for group A and 96.8 % for group B (*p*-value = 0.002). Six of the 12 lesions with false negative results underwent another biopsy, which confirmed adenocarcinoma. Two of the false-negative lesions received wedge resection, which revealed mucinous adenocarcinoma and metastatic endometrial stromal sarcoma. Two patients with false-negative lesions received surgical removal of the brain tumors, which revealed metastatic pulmonary adenocarcinoma. One patient with the false-negative result underwent biopsy of axillary lymph nodes, which revealed metastatic pulmonary adenocarcinoma. Another false-negative lesion was tumor necrosis from the biopsy result, but pulmonary metastatic melanoma was highly suspected based on the melanoma history of the patient.

**Table 1:** Patient characteristics, lesion variables and procedure variables

	Group A (n=43)	Group B (n=155)	*P*-value
Patient variables			
Age (mean ± SD)	63.5 ± 10.6	66.7 ± 12.4	0.124
Sex			
Male	20	86	0.297
Female	23	69	
Lung function test			
Normal	11	21	0.187
Obstructive	7	24	
Restrictive	4	24	
Non-available	21	86	
Lesion variables			
Mean size of the target lesion (mm) (mean ± SD)	12.0 ± 3.2	36.9 ± 18.0	< 0.001
Location of the lesions			
Upper/Middle lobes	25	93	0.826
Lower lobes	18	62	
Distance from pleura (mm) (mean ± SD)	23.2 ± 19.9	16.4 ± 16.3	0.023
Procedure variables			
Patient positions			
Supine	19	69	0.729
Prone	21	81	
Lateral decubitus	3	5	
Number of sampling	2.1 ± 1.1	2.1 ± 0.9	0.835
Complications			
Pneumothorax	22	54	0.051
Chest tube insertion	0	3	1.000
Pulmonary hemorrhage	32	91	0.060
Radiation dose (DLP) (mean ± SD)	1178.6 ± 607.8	1059.2 ± 649.7	0.351
Accuracy	83.7%	96.8%	0.002

*SD* standard deviation, *DLP* dose-length product

**Table 2:** Diagnostic accuracy

Result	Nodule size		*P*-value
	≦15 mm (n = 43)	>15 mm (n = 155)	
True positive	24	113	
True negative	12	37	
False positive	0	0	
False negative	7	5	
Accuracy	83.7 %	96.8 %	0.002

Regarding to the procedural complications, there were 76 cases (38.4 %) of pneumothorax, 3 cases (1.5 %) of pneumothorax with a need for intervention, 123 cases (62.1 %) of pulmonary hemorrhage, 22 cases (11.1 %) of hemoptysis with spontaneous hemostasis, and 3 cases (1.5 %) of hemothorax. No patient suffered from needle track seeding, air embolism or death from the procedure. We also investigated risk factors for post-biopsy pneumothorax and pulmonary hemorrhage. The factors related to post-biopsy pneumothorax are listed in Table 3. The longer needle path length from the pleura to the target lesion (mean ± standard deviation, 21.5 ± 18.3 mm in pneumothorax group versus 15.6 ± 16.5 mm in non-pneumothorax group, *p*-value = 0.020), location in the lower lobes (Odds Ratio [OR]: 2.493, 95 % confidence interval [CI]: 1.382 to 4.498, *p* = 0.002), and obstructive lung function test (compared with normal lung function test, OR: 3.810, 95 % CI: 1.275 to 11.385, *p* = 0.014) were associated with post-biopsy pneumothorax. The factors associated with post-biopsy pulmonary hemorrhage are listed in Table 4. These factors included longer needle path length between pleura and the lesion (mean ± standard deviation, 23.5 ± 17.0 mm in pulmonary hemorrhage group versus 8.6± 13.5 mm in non-pulmonary hemorrhage group, *p*-value < 0.001), smaller target lesion size (mean ± standard deviation, 27.0 ± 15.2 mm in pulmonary hemorrhage group versus 38.8 ± 22.1 mm in non-pulmonary hemorrhage group, *p*-value < 0.001), non-pleural contact lesions (OR: 6.579, 95 % CI: 3.413 to 12.658, *p*-value < 0.001), non-restrictive lung function test (OR: 3.333, 95 % CI: 1.319 to 8.403, *p*-value = 0.009), and patients in supine positions (compared with prone positions, OR: 3.068, 95 % CI: 1.642 to 5.733, *p*-value < 0.001).

**Table 3:** Univariate analysis of selected technique and lesion-related variables as risk factors for pneumothorax

	Pneumothorax (n = 76)	No pneumothorax (n= 122)	*P*-value
Needle path length from pleura to the target (mm) (mean ± SD)	21.5 ± 18.3	15.6 ± 16.5	0.020
Nodule size (mm) (mean ± SD)	28.9 ± 17.9	33.1 ± 19.5	0.134
Target location			0.002
Upper/ middle lobes	35	83	
Lower lobes	41	39	
Number of sampling (mean ± SD)	2.1 ± 1.0	2.1 ± 0.9	0.943
Pleural contact			0.062
Yes	32	68	
No	44	54	
Lung function (If available)			0.034
Normal lung function	7	25	
Obstructive lung function	16	15	
Restrictive lung function	8	20	
Patient postures			0.126
Prone	40	62	
Supine	32	56	
Lateral decubitus	4	4	

*SD* standard deviation

**Table 4:** Univariate analysis of selected technique and lesion-related variables as risk factors for pulmonary hemorrhage

	Pulmonary hemorrhage (n = 123)	No pulmonary hemorrhage (n= 75)	*P*-value
Needle path length from pleura to the target (mm) (mean ± SD)	23.5 ± 17.0	8.6 ± 13.5	< 0.001
Nodule size (mm) (mean ± SD)	27.0± 15.2	38.8 ± 22.1	< 0.001
Target location			0.089
Upper/ middle lobes	79	39	
Lower lobes	44	36	
Number of sampling (mean ± SD)	2.2 ± 1.0	2.0 ± 1.0	0.152
Pleural contact			< 0.001
Yes	42	58	
No	81	17	
Lung function (If available)			0.034
Normal lung function	23	9	
Obstructive lung function	22	9	
Restrictive lung function	12	16	
Patient postures			0.003
Prone	52	50	
Supine	67	21	
Lateral decubitus	4	4	

*SD* standard deviation

## Discussion

The present study showed a significant difference between the diagnostic accuracy of small nodules (less or equal to 15 mm) and larger nodules (larger than 15 mm). The accuracy of the overall biopsy was 93.9 %, and the accuracies for small nodules and larger nodules were 83.7 % and 96.8 %, respectfully. Previous studies [2–16] revealed accuracies for small nodules that ranged from 51.4 % to 95.8 %. The accuracies for smaller lung nodules in previous studies are summarized in Table 5. The high variability may be attributed to several factors, including patient selection, experience of the performer, presence or absence of a bed-side pathologist, and biopsy tools selection. Westcott et al. [6] performed CT-guided aspiration using 20-gauge slotted needles on lesions less than or equal to 15 mm and found an accuracy of 95.3 %. This high accuracy may be partially attributed to the presence of a bed-side pathologist. When the sample was not diagnostic, the pathologist could check the result for the performer immediately, and additional aspiration could be performed if the tissue was non-diagnostic. However, due to the different policies of different hospital, bed-side pathologists are not always present. When the pathologists are not available on-site, the practice of obtaining as much sample tissue as possible is intuitive. Some studies [2, 3] suggested that core biopsies were more suitable for this situation than aspirations. Wallace et al. [8] found an accuracy of 87.7 % in 61 patients with small lung nodules less than or equal to 10 mm. This high accuracy was impressive two decades ago. However, 57 of the 61 patients had pre-existing primary malignancy. The bias of patient selection may partially attribute to the high accuracy, due to the higher recognition rate of malignant tissue under pathological analysis. Kothary et al. [4] performed CT-guided biopsies using 19-gauge guiding needles and 20-gauge biopsy guns on 37 patients with lesions equal to or smaller than 15 mm and found an overall accuracy of 51.4%. The cause of the low accuracy rate was attributed to the benign tendency of small nodules in their study. Pathologists have a more difficult time making a definitive diagnosis when the sample is benign. Choi et al. [3] performed CT-guided biopsies using aspirations or core biopsies on 305 lesions smaller than 10 mm and found an accuracy of 95 %. The study was reviewed, and just 229 of the 305 lesions had a definite correct tissue diagnosis. Because the study design compared the wrongly diagnosed results with correctly diagnosed results, the 27 non-diagnostic results and 37 lost follow-up lesions were not considered as diagnostic failure. They had 241 lesions with a final diagnosis, and 229 of these diagnoses were accurate. Therefore, a 95 % accuracy was obtained. The present study classified non-diagnostic tissue samples as diagnostic failure, which is an important factor the profoundly influences the accuracy. Hiraki et al. [12] performed CT fluoroscopy-guided biopsies using a 19-gauge introducer needle and 20-gauge automated cutting biopsy needle on 1000 lesions. A total of 795 of the 1000 lesions were less than 30 mm. The diagnostic accuracy of the 795 lesions was 95.8 %. This high accuracy was attributed to the novel method of CT fluoroscopy guidance. CT fluoroscopy facilitated the real-time adjustment of the needle trajectory and reduced time-consuming procedures. This study was reviewed, and one attending doctor, or resident doctors under the supervision of the same attending doctor, performed all of the biopsies. The experience of the attending doctor may influence the overall results significantly. However, the CT fluoroscopy-guided procedure is somewhat beyond the discussion of the present study. Further study on this method may be performed in the future. Briefly, the present study found an accuracy of 83.7 % for small nodules. This accuracy is not very high, but acceptable in the absence of an on-site pathologist, and many of the patients had no known history of malignant disease.

**Table 5:** Diagnostic accuracy of small lung nodules using CT-guided techniques in previous studies

Authors	Number of biopsies	Gauge (G) of the needles	Lesion size (mm)	Diagnostic accuracy (%)
Li et al. [23]	27	19-G guiding needles and 22-G aspiration needles	≦ 15	74
Westcott et al. [6]	63	20-G aspiration needles	≦ 15	95.3
Laurent et al. [7]	67	19-G guiding needles and 20-G cutting needles	< 20	91
Tsukada et al. [24]	72	18-G guiding needles and 19-G cutting needles	≦ 20	76.4
Wallace et al. [8]	61	18-G guiding needles and 20- to 22-G aspiration needles	≦ 10	87.7
Ohno et al. [9]	162	22-G aspiration needles	≦ 20	77.2
Shimizu et al. [10]	96	19-G guiding needles and 22-G aspiration needles	< 20	64.6
Ng et al. [11]	55	19-G guiding needles and 22-G aspiration needles	≦ 10	79
Hur et al. [2]	20	20- to 22-G aspiration needles	≦ 20	80
Hiraki et al. [12]	795	19-G guiding needles and 20-G cutting needles	< 30	95.8
Lu et al. [13]	52	19-G guiding needles and 20-G cutting needles	≦30	94
De Filippo et al. [15]	109	22-G aspiration needles	≦ 30	85
Choi et al. [3]	305	20-G cutting or aspiration needles	< 10	95
Li et al. [5]	169	19-G guiding needles and 20-G cutting needles	≦ 20	93.5
Tian et al. [16]	560	17-G guiding needles and 18-G cutting needles	≦ 30	94.6
Huang et al. (present study)	43	17-G guiding needles and 18-G cutting needles	≦ 15	83.7

*G* gauge

Some studies [4, 9, 23] compared the accuracy of small and larger nodules and found significant difference in accuracy. The present study revealed a significant difference in accuracy, and the cause of this difference was quite straightforward. The smaller the nodule, the more difficult it is to obtain adequate tissue for pathological analysis. However, some studies [6–8] found no significant difference in accuracy. Laurent et al. [7] compared nodules equal to or less than 20 mm with nodules larger than 20 mm. The diagnostic accuracies were 91 % and 96.2 % for small and larger nodules, respectfully. No significant difference was found. This result may be attributed to the well-experienced performers and on-site pathologists, which helped achieve these high accuracy rates. Therefore, no difference in accuracy was detected.

The Society of Interventional Radiology (SIR) Guidelines [25] divides complications into minor and major complications. Minor complications include pneumothorax without need for intervention, pulmonary hemorrhage around the target, and hemoptysis with spontaneous hemostasis. Major complications include pneumothorax with a need for intervention, hemothorax, needle tract seeding, air embolism and death. The overall pneumothorax rate in the present study was 38.4 %. Although the instruments were 17-gauge guiding needles, the pneumothorax rate was not particularly high. The pneumothorax rate from previous articles ranged from 15 % to 62 % [4, 6–11, 17, 19, 23, 26, 27]. The high range may be attributed to the different gauges of passing needles, differences in the size of lesions, the use of different techniques, and differences in experience. There are some consensus on preventing pneumothorax, including the use of a coaxial method to prevent multiple passes through the pleura, prevention of crossing the fissure, prevention of crossing of pre-existing blebs or bullae, proper patient selection, and use of blood patch after removal of the introducer needle [28–30]. The factors associated with pneumothorax were analyzed in the present study, and the results demonstrated that needle path length from the pleura to target, location of the lesion, and obstructive lung disease were associated with pneumothorax, which is consistent with previous articles [9, 17, 20, 23, 31]. Other factors mentioned in previous articles include smaller nodules [17, 20], larger numbers of puncture [9], and lesion location in the lower lungs [17]. Insertion of a chest tube is necessary for medial or larger pneumothorax and symptomatic or ongoing increasing size of pneumothorax. Three patients required chest tube insertion, and the percentage of chest tube insertion was 1.5 % in the present study, which is within the reported range of 0%-17% [20, 32–34]. All chest tube insertions occurred in the larger nodule group. However, no significant difference in chest tube insertion rate was found between the small and large nodule groups due to the small number of comparisons.

The pulmonary hemorrhage rate in the present study was 62.1 %. The rate of pulmonary hemorrhage in previous studies ranged from 30 % to 65.6 % [20–22]. The high variety may be attributed to different gauges of passing needle, the use or non-use of the coaxial method, the experience of the performer, and patient selection. The present study defined pulmonary hemorrhage as peri-lesional or peri-needle-path ground glass opacity (GGO) or patchy opacity that was not seen in pre-procedure CT images. The higher incidence rate of pulmonary hemorrhage does not inevitably equal worse outcome for the patients. Tai et al. [22] retrospectively enrolled 1175 cases of lung biopsy to analyze the risk factors associated with pulmonary hemorrhage and found a pulmonary hemorrhage rate of 41.4 %. Only 5 cases with post-biopsy pulmonary hemorrhage had a prolonged hospitalization. The risk factors associated with pulmonary hemorrhage in the present study included longer needle path length, smaller target lesions, non-pleural contact lesions, non-restrictive lung function test, and patients in supine positions. Previous studies [18, 20, 22] reported the same associations, except the protective effect of restrictive lung function test. To explain such finding is difficult, and further study is necessary to confirm it. The hypothesis could be that thickened interlobular septa and fibrosis structures in restrictive lungs reduce the possibility of needles piercing the vessels. Other factors associated with post-biopsy pulmonary hemorrhage in previous studies [18, 20, 22] are lower zone located lesions, female, older age and emphysema. These factors were analyzed in the study, but no significant difference was found.

### Study limitations

This study was a single-center and retrospective study and the case number is relatively small. The performers of the biopsy procedures had experience of 3 years to 26 years. The variation in experience may have also influenced the overall study results. The pneumothorax and pulmonary hemorrhage were deemed present or absent. Further quantization of pneumothorax and pulmonary hemorrhage on CT images could be performed in further studies.

## Conclusion

In conclusion, CT-guided biopsies of small nodules equal to or less than 15 mm using 17-gauge guiding needles and 18-gauge biopsy guns are accurate and safe. The biopsy results of small lesions were less accurate than the results of large lesions, but the results provided a reliable reference for clinical decision-making. The factors associated with post-biopsy pneumothorax in the present study were longer needle path length between pleura and the lesion, lesion location in the lower lobes, and patients with obstructive lung function test. The factors associated with post-biopsy pulmonary hemorrhage in the study were longer needle path length between pleura and the lesion, smaller target lesions, non-pleural contact lesions, non-restrictive lung function test, and patients in supine positions. An understanding of these factors may facilitate pre-procedure planning. Physicians should be aware of patients with risk factors and communicate these risks to the patient and family comprehensively.

## Data Availability

The datasets used and/or analyzed during the current study are available from the corresponding author on reasonable request.

## Abbreviations

CT: Computed tomography
DLP: Dose-length product
EBB: Endobronchial biopsies
FNAB: Fine needle aspiration biopsy
G: Gauge
GGO: Ground glass opacity
IRB: Institutional review board
PT-INR: Prothrombin time-to-international normalized ratio
SD: Standard deviation
SIR: Society of interventional radiology
VATS: Video-assisted thoracoscopy

## References

[1] Siegel RL, Miller KD, Jemal A (2018). Cancer statistics, 2018. CA Cancer J Clin..

[2] Hur J, Lee HJ, Nam JE, Kim YJ, Kim TH, Choe KO (2009). Diagnostic accuracy of CT fluoroscopy-guided needle aspiration biopsy of ground-glass opacity pulmonary lesions. AJR Am J Roentgenol..

[3] Choi SH, Chae EJ, Kim JE, Kim EY, Oh SY, Hwang HJ (2013). Percutaneous CT-guided aspiration and core biopsy of pulmonary nodules smaller than 1 cm: analysis of outcomes of 305 procedures from a tertiary referral center. AJR Am J Roentgenol..

[4] Kothary N, Lock L, Sze DY, Hofmann LV (2009). Computed tomography-guided percutaneous needle biopsy of pulmonary nodules: impact of nodule size on diagnostic accuracy. Clin Lung Cancer..

[5] Li Y, Du Y, Yang HF, Yu JH, Xu XX (2013). CT-guided percutaneous core needle biopsy for small (≤20 mm) pulmonary lesions. Clin Radiol..

[6] Westcott JL, Rao N, Colley DP (1997). Transthoracic needle biopsy of small pulmonary nodules. Radiology..

[7] Laurent F, Latrabe V, Vergier B, Montaudon M, Vernejoux JM, Dubrez J (2000). CT-guided transthoracic needle biopsy of pulmonary nodules smaller than 20 mm: results with an automated 20-gauge coaxial cutting needle. Clin Radiol..

[8] Wallace MJ, Krishnamurthy S, Broemeling LD, Gupta S, Ahrar K, Morello FA (2002). CT-guided percutaneous fine-needle aspiration biopsy of small (< or =1-cm) pulmonary lesions. Radiology..

[9] Ohno Y, Hatabu H, Takenaka D, Higashino T, Watanabe H, Ohbayashi C (2003). CT-guided transthoracic needle aspiration biopsy of small (< or = 20 mm) solitary pulmonary nodules. AJR Am J Roentgenol..

[10] Shimizu K, Ikeda N, Tsuboi M, Hirano T, Kato H (2006). Percutaneous CT-guided fine needle aspiration for lung cancer smaller than 2 cm and revealed by ground-glass opacity at CT. Lung Cancer..

[11] Ng YL, Patsios D, Roberts H, Walsham A, Paul NS, Chung T (2008). CT-guided percutaneous fine-needle aspiration biopsy of pulmonary nodules measuring 10 mm or less. Clin Radiol..

[12] Hiraki T, Mimura H, Gobara H, Iguchi T, Fujiwara H, Sakurai J (2009). CT fluoroscopy-guided biopsy of 1,000 pulmonary lesions performed with 20-gauge coaxial cutting needles: diagnostic yield and risk factors for diagnostic failure. Chest..

[13] Lu CH, Hsiao CH, Chang YC, Lee JM, Shih JY, Wu LA (2012). Percutaneous computed tomography-guided coaxial core biopsy for small pulmonary lesions with ground-glass attenuation. J Thorac Oncol..

[14] Inoue D, Gobara H, Hiraki T, Mimura H, Kato K, Shibamoto K (2012). CT fluoroscopy-guided cutting needle biopsy of focal pure ground-glass opacity lung lesions: diagnostic yield in 83 lesions. Eur J Radiol..

[15] De Filippo M, Saba L, Concari G, Nizzoli R, Ferrari L, Tiseo M (2013). Predictive factors of diagnostic accuracy of CT-guided transthoracic fine-needle aspiration for solid noncalcified, subsolid and mixed pulmonary nodules. Radiol Med..

[16] Tian P, Wang Y, Li L, Zhou Y, Luo W, Li W (2017). CT-guided transthoracic core needle biopsy for small pulmonary lesions: diagnostic performance and adequacy for molecular testing. J Thorac Dis..

[17] Nour-Eldin NE, Alsubhi M, Emam A, Lehnert T, Beeres M, Jacobi V (2016). Pneumothorax Complicating Coaxial and Non-coaxial CT-Guided Lung Biopsy: Comparative Analysis of Determining Risk Factors and Management of Pneumothorax in a Retrospective Review of 650 Patients. Cardiovasc Intervent Radiol..

[18] Nour-Eldin NE, Alsubhi M, Naguib NN, Lehnert T, Emam A, Beeres M (2014). Risk factor analysis of pulmonary hemorrhage complicating CT-guided lung biopsy in coaxial and non-coaxial core biopsy techniques in 650 patients. Eur J Radiol..

[19] Heerink WJ, de Bock GH, de Jonge GJ, Groen HJ, Vliegenthart R, Oudkerk M (2017). Complication rates of CT-guided transthoracic lung biopsy: meta-analysis. Eur Radiol..

[20] Yeow KM, Su IH, Pan KT, Tsay PK, Lui KW, Cheung YC (2004). Risk factors of pneumothorax and bleeding: multivariate analysis of 660 CT-guided coaxial cutting needle lung biopsies. Chest..

[21] Freund MC, Petersen J, Goder KC, Bunse T, Wiedermann F, Glodny B (2012). Systemic air embolism during percutaneous core needle biopsy of the lung: frequency and risk factors. BMC Pulm Med..

[22] Tai R, Dunne RM, Trotman-Dickenson B, Jacobson FL, Madan R, Kumamaru KK (2016). Frequency and Severity of Pulmonary Hemorrhage in Patients Undergoing Percutaneous CT-guided Transthoracic Lung Biopsy: Single-Institution Experience of 1175 Cases. Radiology..

[23] Li H, Boiselle PM, Shepard JO, Trotman-Dickenson B, McLoud TC (1996). Diagnostic accuracy and safety of CT-guided percutaneous needle aspiration biopsy of the lung: comparison of small and large pulmonary nodules. AJR Am J Roentgenol..

[24] Tsukada H, Satou T, Iwashima A, Souma T (2000). Diagnostic accuracy of CT-guided automated needle biopsy of lung nodules. AJR Am J Roentgenol.

[25] Gupta S, Wallace MJ, Cardella JF, Kundu S, Miller DL, Rose SC (2010). Quality improvement guidelines for percutaneous needle biopsy. J Vasc Interv Radiol..

[26] Ohno Y, Hatabu H, Takenaka D, Imai M, Ohbayashi C, Sugimura K (2004). Transthoracic CT-guided biopsy with multiplanar reconstruction image improves diagnostic accuracy of solitary pulmonary nodules. Eur J Radiol..

[27] Lucidarme O, Howarth N, Finet JF, Grenier PA (1998). Intrapulmonary lesions: percutaneous automated biopsy with a detachable, 18-gauge, coaxial cutting needle. Radiology..

[28] Wagner JM, Hinshaw JL, Lubner MG, Robbins JB, Kim DH, Pickhardt PJ (2011). CT-guided lung biopsies: pleural blood patching reduces the rate of chest tube placement for postbiopsy pneumothorax. AJR Am J Roentgenol..

[29] Malone LJ, Stanfill RM, Wang H, Fahey KM, Bertino RE (2013). Effect of intraparenchymal blood patch on rates of pneumothorax and pneumothorax requiring chest tube placement after percutaneous lung biopsy. AJR Am J Roentgenol..

[30] Clayton JD, Elicker BM, Ordovas KG, Kohi MP, Nguyen J, Naeger DM (2016). Nonclotted blood patch technique reduces pneumothorax and chest tube placement rates after percutaneous lung biopsies. J Thorac Imaging..

[31] Takeshita J, Masago K, Kato R, Hata A, Kaji R, Fujita S (2015). CT-guided fine-needle aspiration and core needle biopsies of pulmonary lesions: a single-center experience with 750 biopsies in Japan. AJR Am J Roentgenol..

[32] Covey AM, Gandhi R, Brody LA, Getrajdman G, Thaler HT, Brown KT (2004). Factors associated with pneumothorax and pneumothorax requiring treatment after percutaneous lung biopsy in 443 consecutive patients. J Vasc Interv Radiol..

[33] Hiraki T, Mimura H, Gobara H, Shibamoto K, Inoue D, Matsui Y (2010). Incidence of and risk factors for pneumothorax and chest tube placement after CT fluoroscopy-guided percutaneous lung biopsy: retrospective analysis of the procedures conducted over a 9-year period. AJR Am J Roentgenol..

[34] Geraghty PR, Kee ST, McFarlane G, Razavi MK, Sze DY, Dake MD (2003). CT-guided transthoracic needle aspiration biopsy of pulmonary nodules: needle size and pneumothorax rate. Radiology..

